# Routine testing for IgG antibodies against hepatitis A virus in Israel

**DOI:** 10.1186/1471-2458-5-60

**Published:** 2005-06-06

**Authors:** Noah Samuels

**Affiliations:** 1 Maccabi Healthcare Services, 130 Rachmilevich Street, Jerusalem 97791, Israel

## Abstract

**Background:**

Viral hepatitis is highly endemic in Israel, with the hepatitis A virus (HAV) responsible for most cases. Improved socioeconomic factors, as well as the universal vaccination of infants (introduced in 1999) has resulted in a decline in infection rates in Israel. This study examines the benefits of routine testing for anti-HAV IgG in high-risk population.

**Methods:**

A retrospective examination of the files of teenage and adult patients (aged 16–99 years; mean 33.9) in two primary care clinics found 1,017 patients who had been tested for anti-HAV IgG antibodies for either general healthcare screening or ongoing follow-up for chronic illness. Seropositive patients were then asked regarding recall of past hepatitis (i.e. jaundice, regardless of viral etiology); post-exposure prophylaxis with immune serum immunoglobulin (ISG); and active immunization with inactivated virus. Seronegative patients were subsequently sent for active immunization.

**Results:**

Of the1,017 patient records studied (503 male, 514 female), a total of 692 were seropositive (354 males, 338 females; P = 0.113). Seropositivity rates increased with age (p < 0.005), and were highest among those born in Middle Eastern countries other than Israel (91.3%) and lowest among immigrants from South America (44.1%; P < 0.005). 456 of the seropositive patients were interviewed, of whom only 91 recalled past illness while 103 remembered receiving post-exposure prophylaxis (ISG) and 8 active vaccination. Those who were unaware of past infection were more likely to have been vaccinated with ISG than those who were aware (26.3% vs. 7.7%; p < 0.005).

**Conclusion:**

The relatively high prevalence rate of anti-HAV seropositivity in our study may me due to the fact that the study was conducted in a primary care clinic or that it took place in Jerusalem, a relatively poor and densely populated Israeli city. Most of the seropostive patients had no recollection of prior infection, which can be explained by the fact that most hepatitis A infections occur during childhood and are asymptomatic. Routine testing for anti-HAV IgG in societies endemic for HAV would help prevent seropositive patients from receiving either post-exposure or preventive immunization and target seronegative patients for preventive vaccination.

## Background

For years Israel has been considered to be endemic for viral hepatitis, with an incidence of reported cases five to ten times that observed in the United States [1]. Most cases of viral hepatitis in Israel are caused by the hepatitis A virus (HAV), and in the 1970's the Israeli Ministry of Health instituted a wide-scale program of post-exposure prophylaxis with immune serum globulin (ISG). In 1999 universal immunization with inactivated virus (Havrix; Smith Klein Beecham) was instituted for infants, the first dose being given at 18 months and the second at 24 – 30 months of age [2]. The Israel Defense Forces (IDF) have instituted vaccination programs as well, first with ISG and, more recently, with the inactivated virus [3].

Vaccination programs, as well socioeconomic changes, have led to a significant reduction in the prevalence of HAV infection in Israel today [4,5]. In the United States, the U.S. Advisory Committee on Immunization Practices has given clear recommendations for vaccinating communities with high rates of infection (>20 cases per 100,000) or populations at risk for liver failure [6]. However, no guidelines exist for routine testing for HAV serology prior to vaccination, a policy which could potentially prevent unnecessary immunizations (both active and passive) in seropositive patients as well as target those who are seronegative for active immunization. The following study examined the current prevalence of anti-HAV serology in a selected Israeli population (patients presenting to a primary care clinic), evaluating the benefits of routine testing for populations at risk.

## Methods

The records of teenage and adult patients (aged 16–99 years; mean 33.9) from two primary care clinics in Jerusalem, Israel, were retrospectively searched for anti-HAV IgG serology. The clinics are affiliated with the Maccabi Health Care Services, one of four government-funded services available to all Israeli citizens. Patients had undergone general blood tests for a number of reasons, either for follow-up of existing illness (none of which were hepatic-related) or for routine health screening, including anti-HAV IgG serology. A total of 1,017 patient files were found which contained serological results of anti-HAV antibodies from blood analyzed in the health fund's central lab, which used a microparticle enzymatic assay (MEA; AxSYM System, Abbott Diagnostics). Patients who tested seropositive for HAV were then contacted (either in person or by phone) and asked regarding the following: demographic data (country of birth); whether or not they remembered being sick with hepatitis (i.e. clinical jaundice, with or without known serological evidence of active HAV infection), and when; and whether or not they had been immunized, either passively (ISG) or actively (inactivated virus), and when. Recall of past infection was based on patient reporting alone and not through review of medical or laboratory records. Data was compiled and analyzed using the Microsoft Excel program.

## Results

### Prevalence of HAV seropositivity

**Figure 1:**
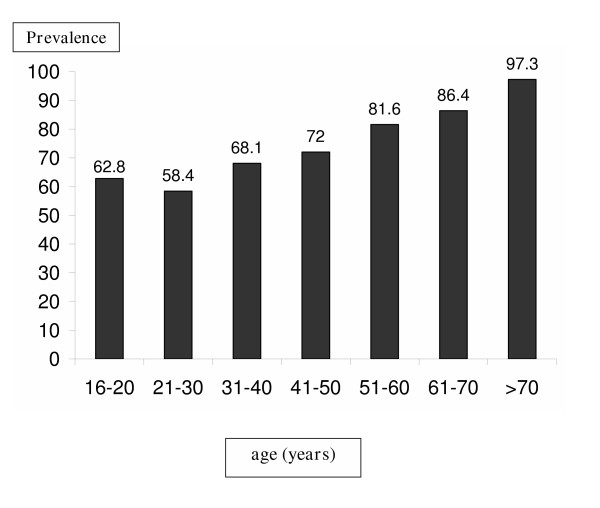
Prevalence of positive HAV serology according to age (%)

As mentioned above, a total of 1,017 patient records (503 males and 514 females) were found to contain results of anti-HAV antibodies (from March 1998 to September 2003). The mean age of the study group was 33.9 years; 37.8 among males (range: 16–99) and 31.2 among females (range: 16–87). Among the recorded results, 692 (68.0%) were seropositive – 354 of the male patients (70.4%) and 338 of the female patients (65.8%; p = 0.113). The rates of seropositivity increased with age (see Figure 1; p < 0.005), and remained constant throughout the years of the study (p = 0.451).

**Figure 2:**
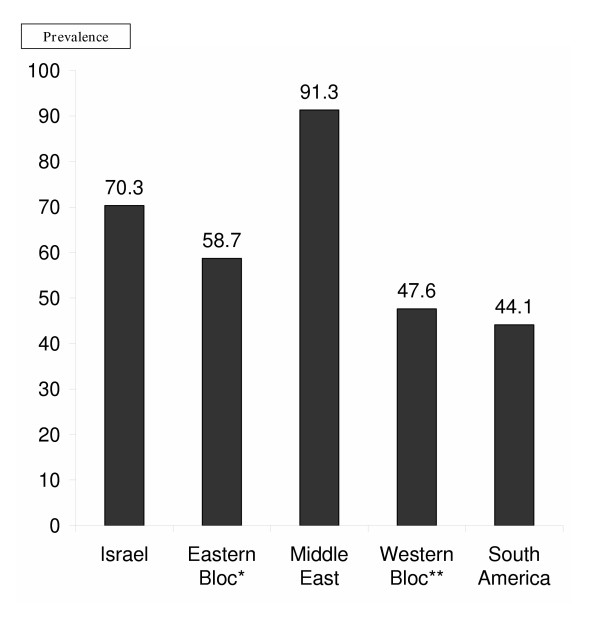
**Prevalence of positive HAV serology according to country of birth(%)**. * **Eastern Bloc **= former USSR and Eastern European countries** **Western Bloc **= North America and Western Europe

Patients who were born in Middle Eastern countries other than Israel had the highest seropositive rate (91.3%), while those born in South America the lowest (44.1%; p < 0.005. See Figure 2). Immigrants from the Eastern Bloc countries (former Soviet Bloc and Eastern Europe) had a significantly higher prevalence of anti-HAV antibodies than native Israelis (70.3% vs. 58.7%; p = 0.016).

### Recall of past infection and immunization

Of the 658 patients with anti-HAV antibodies, 456 were interviewed and asked regarding past infection and vaccinations (231 males and 225 females). Only 91 had any recollection of prior illness (20.0%) – 51 of the males interviewed (22.1%) and 40 of the females (17.8%; p = 0.249), all of them at least 5 years prior to undergoing the serological testing. A total of 103 respondents remembered receiving post-exposure prophylaxis (passive immunization) with ISG. Those who were unaware of past infection were more likely to have been vaccinated with ISG than those who were aware (26.3% vs. 7.7%; p < 0.005). The reasons given for receiving ISG are listed in Table 1. Eight patients (2 male, 6 female) reported receiving the inactivated virus vaccine, of which none were aware of prior illness.

**Table 1:** Reasons for Passive Immunization* in Seropositive Patients

**Reason**	**Male (n)**	**Female**	**Total**
**Post-exposure prophylaxis**	25	25	50
**Travel to endemic country**	10	3	13
**Army Service**	14	3	17
**Other**	17	6	23
**Total**	66	37	103

* passive immunization = immune serum globulin

## Discussion

Although Israel is considered endemic for HAV infection, the prevalence of anti -HAV antibodies in our study population (68%) is much higher than that found in other studies, especially among the under-20 age group (62.8%). In a 1996 study of teenagers undergoing pre-draft evaluation, only 38.4% were seropositive [5], while serum samples from the Israeli Center for Disease Control's national serum bank in 1989 were seropositive for HAV in only 27% of 16 year-olds [4]. The relatively high prevalence of anti-HAV antibodies found in our study may be due to the fact that the study was conducted in a primary care clinic, with higher morbidity rates than the general population, though not liver-related. Another factor is the fact that both clinics are located in Jerusalem, one of Israel's poorest and most densely populated cities. Many of the patients were ultra-orthodox Yeshiva students who are at greater risk for HAV infection [7] and are not drafted into the IDF. At the same time, the higher rate of seropositivity among immigrants from Eastern bloc countries is consistent with the findings of an earlier study of this population [8], though another study of pre-draft teenagers did not find a significant difference between these immigrants and native Israelis [9].

Most of the seropositive patients in our study (80%) had no prior recollection of HAV infection. For 70% of children under the age of 6 (in communities with high rates of hepatitis A, 30–40% of children acquire infection before the age of 5 6) HAV infection is a self-limited, subclinical disease [10]. Among older children and adults, infection is usually symptomatic, with jaundice occurring in more than 70% of patients [11]. Signs and symptoms usually last less than 2 months, although 10–15% of symptomatic persons have prolonged or relapsing disease lasting up to 6 months [12]. Case fatality rates also increase with age, rising from 1.5/1000 among children less than 5 years old to 27/1000 after age 50 [13], making vaccination of unimmunized adults that much more important.

Both ISG and inactivated HAV vaccines are safe, though not without potential side effects both locally and systemically [6,14]. Aside from having to undergo two injections within a span of 6 months to a year, adult patients in Israel are required to purchase the active vaccine themselves. Seropositive patients in our study who were unaware of their immune status were more likely (by a factor of 3.4) to have unnecessarily received ISG (unnecessarily since they most likely have lifetime immunity) than those who were aware of past infection. This is probably an underestimate, since both post-exposure and pre-induction vaccinations have been implemented in Israel for nearly thirty years, and it is likely that many have forgotten receiving immunizations in the distant past. For the eight patients who received the inactivated virus without prior serological testing, it is possible (and even probable) that they too were immunized unnecessarily. Now it is too late to know whether their current seropositive status is a result of past occult infection (with lifetime immunity) or active immunization. These patients will now have to receive periodic booster vaccinations, which may have been unnecessary if prior immune status had been checked. Testing for HAV serology prior to active immunization has been shown to be both cost-effective and economically valid for Israeli travelers, assuming a cost of $130 for vaccination (which is subsidized but not free for the patient) and $30 for the IgG test (free for the patient) [15]. This may be true for the general population as well.

## Conclusion

The recent adoption of a nationwide infant HAV immunization policy in Israel has also been found to be both medically and economically justifiable [16]. However, this program is aimed at the infant population, for the purpose of preventing early childhood infection. Screening the adult population in endemic countries like Israel would enable seronegative adults to receive the inactivated virus, thus preventing morbidity and mortality resulting from infection. For the seropositive adult population, unnecessary immunizations could be avoided. Asking patients whether or not they need to be vaccinated is, at best, unreliable, since most cases of HAV infection occur in childhood and are asymptomatic, and even in cases where jaundice is present the cause may not necessarily be HAV. The health establishment in Israel needs to investigate the benefits of routing testing for anti-HAV serology, especially among the high-risk adult population who were not actively immunized during childhood or military service. Such routine testing is feasible, especially in an era where it is accepted practice to conduct routine screening tests for a number of preventive measures.

## Competing interests

The author(s) declare that they have no competing interest.

## Authors' contributions

The design and implementation of the study, as well as statistical analysis of the data, were all done by the author (NS).

## Pre-publication history

The pre-publication history for this paper can be accessed here:


